# 

**Published:** 1893-03

**Authors:** 


### CONTENTS.

CLINICAL LECTURE—
Surgical Clinic at Harlem Hospital, New York. By Thomas H. Manley, M. D.... 1
ORIGINAL COMMUNICATIONS—
On Certain Animal Extracts—Their Mode of Preparation and Physiological and Therapeutical Effects. By William Hammond, M. D., Washington, D. C.. 5
Some Comparatively New Facts About an Old Remedy—The Muriated Tincture of Iron. By E. F. Starr, M. D., Nacoochee, Ga.......... 14
Uterine Fibroma. By J. B. Murfree, A. M., M. D., Murfreesboro, Tenn.......... 18
SOCIETY REPORTS—
Clinical Society of Maryland.......... 20
CORRESPONDENCE—
New York Letter.......... 26
Case of Cretinism. Hugh Hagan, M. D., Atlanta, Ga.......... 31
EDITORIAL—
The New Volume.......... 33
We Thank You, Gentlemen.......... 34
An Eclectic Reviewer.......... 35
The Pan American Medical Congress.......... 37
Medical College Commencements.......... 38
SELECTIONS AND ABSTRACTS,
General Medicine and Therapeutics.......... 40
Surgery.......... 46
Obstetrics and Gynecology.......... 50
Skin and Genito-Urinary Diseases.......... [illegible]
MEDICAL ITEMS.......... 59
BOOK REVIEWS.......... 62
INDEX TO ADVERTISEMENTS.......... xii
ITEMS OF INTEREST.......... x xi xxxiii xxxiv
PREMIUM OFFERS.......... xv

